# lncRNA-AC079061.1/VIPR1 axis may suppress the development of hepatocellular carcinoma: a bioinformatics analysis and experimental validation

**DOI:** 10.1186/s12967-022-03573-7

**Published:** 2022-08-29

**Authors:** Xia-Hui Lin, Dan-Ying Zhang, Zhi-Yong Liu, Wen-qing Tang, Rong-Xin Chen, Dong-ping Li, Shuqiang Weng, Ling Dong

**Affiliations:** 1 Department of Gastroenterology and Hepatology, Zhongshan Hospital, Fudan University, Shanghai, 200032 China; 2 Shanghai Institute of Liver Disease, Shanghai, 200032 China; 3 Liver Cancer Institute, Zhongshan Hospital, Fudan University and Key Laboratory of Carcinogenesis and Cancer Invasion, Ministry of Education, Shanghai, 200032 China

**Keywords:** Hepatocellular carcinoma (HCC), ceRNA mechanism, Bioinformatics analysis, Proliferation, Invasion, Prognosis

## Abstract

**Background:**

Hepatocellular carcinoma (HCC) is one of the most malignant tumors to threaten human life, and the survival rate remains low due to delayed diagnosis. Meanwhile, lncRNAs have great potential for application in tumor prognosis, therefore relevant research in hepatocellular carcinoma is indispensable.

**Methods:**

Based on the EZH2 expression, the differentially expressed lncRNAs DElncRNAs), miRNAs (DEmiRNAs), and mRNAs (DEmRNAs) were identified in hepatocellular carcinoma by using the TCGA database. Bioinformatics technology was utilized to determine the effect of key genes in HCC progression. The methylation and immune infiltration analyses were performed to explore the underlying function of hub genes. Finally, cellular function experiments were performed to investigate the association between identified genes and biological phenotypes in HCC.

**Results:**

lncRNA-AC079061.1, hsa-miR-765, and VIPR1 were identified as independent factors that affect the prognosis of hepatocellular carcinoma. The immune infiltration analyses revealed that lncRNA-AC079061.1 can alter the immune microenvironment and thus inhibit the development of HCC by regulating the expression of an immune-related gene (VIPR1). Methylation analyses demonstrated that VIPR1 expression is negatively related to the methylation level in HCC. Experimental results suggested that lncRNA-AC079061.1 and VIPR1 were frequently downregulated in HCC cells, while hsa-miR-765 was significantly upregulated. Moreover, the lncRNA-AC079061.1/VIPR1 axis suppressed the proliferation and invasion of HCC cells.

**Conclusion:**

The present study identified the lncRNA-AC079061.1/VIPR1 axis as a novel biomarker that inhibited the proliferation and invasion of hepatocellular carcinoma, affecting the ultimate disease outcome.

**Supplementary Information:**

The online version contains supplementary material available at 10.1186/s12967-022-03573-7.

## Background

Hepatocellular carcinoma (HCC) is the second leading cause of cancer-related mortality worldwide and is commonly seen in patients with chronic liver inflammation associated with viral infection or metabolic syndrome [1]. It accounts for about 90% of non-metastatic liver tumors, which may result in systemic manifestations [2, 3]. Over the past decades, patients have benefited from advancements in new treatment strategies. However, the overall survival rate remains low due to delayed diagnosis of the diseases.

In recent years, the use of statistical models and bioinformatics in identifying novel biomarkers has been increasing [4]. Therefore, such research provided great potential for clinical application. Various studies showed that lncRNAs play a crucial role in various diseases including cancer [5]. For instance, Ma et al. found that EGFR1-mediated lnc01503 can promote gastric cancer progression [6]. While Fan et al. reported that MKL1-induced lncRNA SNHG18 drives the growth and metastasis of non-small cell lung cancer [7].

Enhancer of zeste homolog 2 (EZH2) is a member of the polycomb group genes (PcGs) family, which is an important epigenetic regulator [8]. It has been identified as a common oncogene in various types of tumors and is involved in many biological processes including cell proliferation [9], invasion [10, 11], metabolism [12, 13], and apoptosis [14]. Besides, EZH2 has also been identified as an oncogene in HCC. Chen et al. verified that EZH2 can promote the development of HCC through modulating the miR-22/galectin-9 axis. Therefore, we speculate that the EZH2-related gene can affect the progression of HCC.

In the present study, the differentially expressed lncRNAs/miRNAs/mRNAs are screened based on the EHZ2 expression. Then, the prognostic genes were selected according to the overall survival curve. Multiple databases were used to analyze the possible interaction between lncRNA, miRNA, and mRNA. The corresponding statistical models (cox regression analysis and nomogram) were established to evaluate the survival prediction function of key genes. Methylation and immune infiltration analyses were used to determine the potential function of VIPR1. Finally, PCR and a series of cellular function experiments were performed to confirm the comprehensive analysis. The prediction of hub gene-related drugs can help identify potential treatment targets for HCC.

## Materials and methods

### Data source

**Fig. 1:**
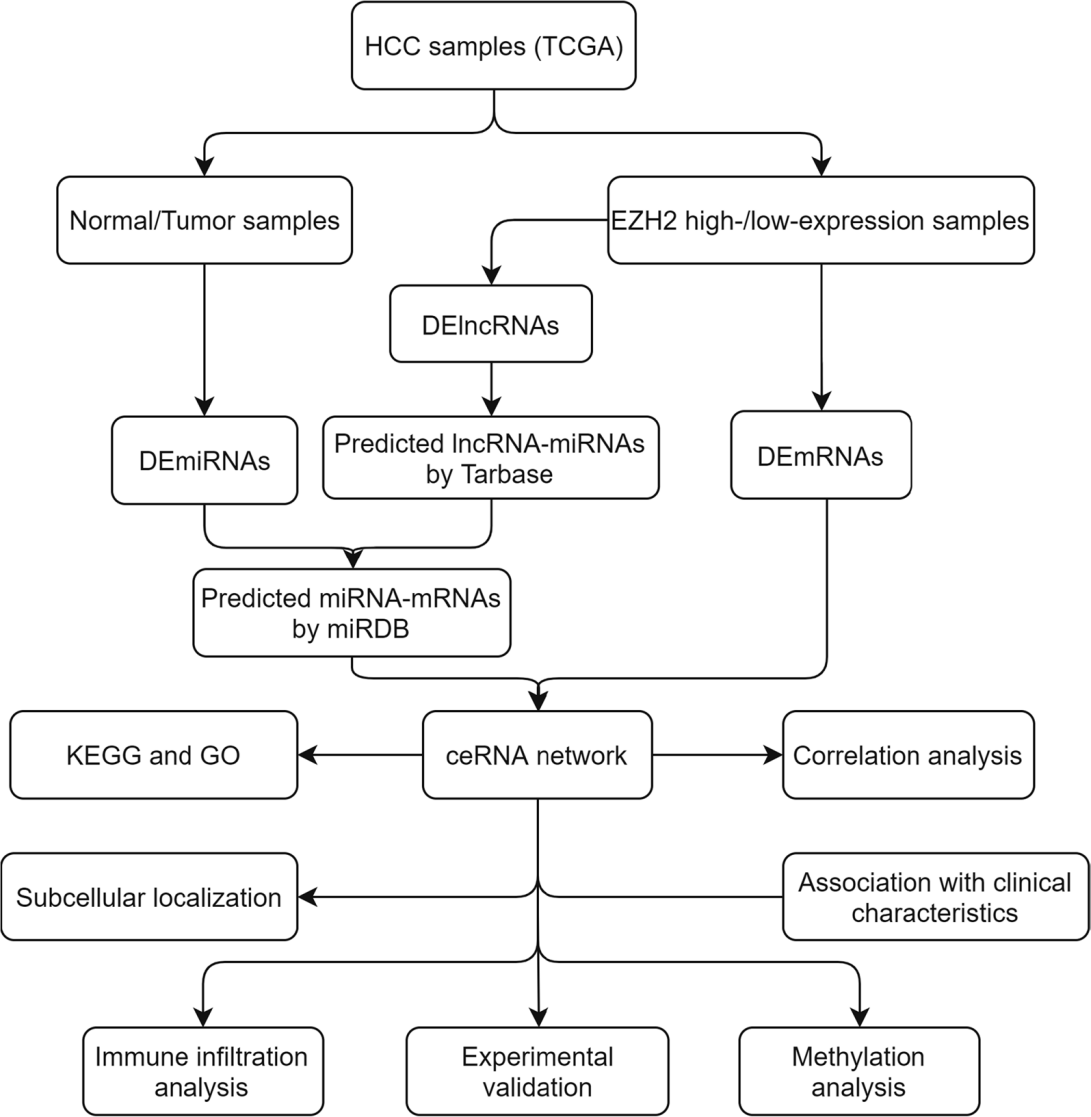
The whole workflow chart of the study

The whole workflow chart of the present study was depicted in Fig. 1. The RNA-seq in TPM (transcripts per million reads) format, miRNA-seq in RPM (reads per million mapped reads) format, and clinical data of hepatocellular carcinoma cases were downloaded from The Cancer Genome Atlas (TCGA) portal (http://cancergenome.nih.gov). The whole cohort consisted of 374 HCC samples and 50 normal samples. The GTEX data were obtained from UCSC XENA (https://xenabrowser.net/datapages/) database. The gene annotation files were downloaded from the GENCODE database and used to extract the lncRNA and mRNA expression matrix, respectively. The immune gene list was obtained from the ImmPort Portal database (https://www.immport.org/home). The targets of lncRNA were predicted from the LncBase v.2 (http://carolina.imis.athena-innovation.gr/diana_tools/web/index.php?r=lncbasev2%2Findex), and the targets of miRNAs were predicted from the miRDB (http://mirdb.org/). The copy-number alteration data was downloaded from the cBioPortal database (http://www.cbioportal.org/), and the immunohistochemistry data were obtained from the Human Protein Atlas (HPA) database (https://www.proteinatlas.org/).

### Differential expression analysis

The RNA-seq were divided into high and low groups according to the EZH2 expression levels. The difference analysis was performed using the “DESeq2” [15]. For better and effective screening of interaction networks, lncRNAs with |logFC|> 2, adjusted p-value < 0.05, miRNAs with |logFC|> 0.5, adjusted p-value < 0.05, and mRNAs with |logFC|> 1, adjusted p-value < 0.05 were considered as differentially expressed genes. All the expression data were log2 transformed.

### Correlation analysis

Since the expression data of RNA-seq and miRNA-seq did not conform to a normal distribution, we utilized the Spearman correlation to analyze the association between lncRNAs, miRNAs, and mRNAs. The results of the Spearman correlation were visualized using the “ggplot2” R package.

### Functional enrichment analysis

To assess the potential mechanisms of the lncRNA-miRNA-mRNA axis, the KEGG and GO enrichment analyses were performed by using the “clusterProfiler” R package [16]. Biological process terms, cellular component terms, molecular function terms, and pathways with adjusted p-value < 0.05 were considered as potential mechanisms for the onset of HCC.

### Immune infiltration analysis

The markers of 24 immune cells were derived from an immunity article, and the classification and description of specific cells can be found in this article [17]. The immune infiltration analysis was performed through the ssGSEA algorithm in the “GSVA” R package [18]. The Pearson correlation analysis was used to evaluate the correlation between immune cells and differentially expressed genes.

### Methylation analysis

The methylation of hub gene in HCC and normal tissues was evaluated through the MEXPRESS (https://mexpress.be/) and MethSurv database (https://biit.cs.ut.ee/methsurv/). The search terms were VIPR1 and liver hepatocellular carcinoma.

### Prognostic analysis

According to the clinical data of HCC, the effects of hub genes on patients’ prognoses were assessed through the Kaplan–Meier curve. The univariate Cox regression analysis was performed to screen for factors to construct the nomogram model. Nomograms were used to evaluate the role of hub genes in HCC through the “rms” and “survival” R packages. P-value < 0.05 was considered significantly.

### Cell lines and cell culture

Human liver cells L0-2, HCC cells (PLC/PRF/5, HepG2, Hep3B) (Cell Bank of the Chinese Academy of Sciences, Shanghai, China), MHCC97H and HCCLM3 (Liver Cancer Institute, Fudan University, Shanghai, China), and Huh7 (Japanese Cancer Research Resources Bank) were cultured in Dulbecco's modified Eagle medium (Gibco) with 10% fetal bovine serum (Gibco) and 1% penicillin–streptomycin (Invitrogen). Cell cultures were done in a thermostatic incubator at 37 °C with a humidified atmosphere of 95% air and 5% CO_2_.

### Transfection experiments

The lncRNA-AC079061.1 small interfering RNA (siRNA) and negative control were purchased from RiboBio Company (Guangzhou, China). The transfection of the lncRNA-AC079061.1 siRNA and negative control was performed with Lipofectamine™ 3000 (#L3000008, ThermoFisher, China) according to the manufacturer’s instructions.

### RNA extraction and quantitative reverse-transcription polymerase chain reaction (qRT-PCR)

The total RNA including miRNA was extracted from cells or tissues using the TRIzol Reagent (Invitrogen), while cDNA was synthesized from RNA using the Reverse Transcription Kit (Takara). Subsequently, cDNA was amplified using the Maxinma SYBR Green qPCR Master Mix (Thermo Scientific). The quantification of target genes was done with the 2^−ΔΔCt^ method using glyceraldehyde-3-phosphate dehydrogenase (GAPDH) for normalization. The primers for hsa-miR-765 and U6 small nuclear RNA were obtained from RiboBio Company (Guangzhou, China). The sequences were covered by a patent. Analyses of miRNA expression were normalized to the expression of internal control U6 using the 2 − ΔΔCT method. Melting curve analysis was carried out to assess the specificity of PCR products. The primers have been used for real time PCR, lncRNA-AC079061.1 Sequence 5′-3′: Forward, CCCTCAGGCATCCACTCTACCC; Reverse, TCCAACGCCACCCACTTCAAAC; VIPR1 Sequence 5′-3′: Forward, ACAAGGCAGCGAGTTTGGATGAG; Reverse, GTGCAGTGGAGCTTCCTGAACAG.

### Luciferase Reporter Assay

MHCC97H cells and HCCLM3 cells were seeded into 96-well plates and co-transfected with a mixture of 60 ng luciferase, 6 ng pRL-CMV Renilla luciferase reporter, and miR-765 mimic or the negative control using the Lipofectamine 2000 transfection reagent (RiboBio, Guangzhou, China), according to the manufacturer’s instructions.). After 48 h of incubation, the firefly and Renilla luciferase activities were measured using a dual-luciferase reporter assay (Promega, Madison, WI, USA).

### Western blot

As described, the protein was extracted from cells or tissues using RIPA cell lysis with Protease Inhibitor Cocktail. Mitochondrial protein extraction was performed with the use of a mitochondrial isolation kit (Beyotime Biotechnology), in accordance with the manufacturer’s directions. The proteins were quantified by using the BCA kit, subjected to 12% SDS-PAGE for separation, then transferred to 0.45 μM PVDF membranes (Millipore, USA). The membranes were blocked with skimmed milk and incubated with primary antibodies at 4 ℃ overnight, followed by incubation with the corresponding HRP-conjugated secondary antibody (PeproTech). The bands were visualized by enhanced chemiluminescence. The intensity of protein expression was measured using ImageJ software. The antibodies used for western blot were listed as follows: VIPR1, Bioss, # bs-2982R, 1:1000; GAPDH, Cell Signaling Technology, #5174, 1:1000.

### CCK-8 and colony formation assays

The cell proliferation assays were performed using a CCK-8 Kit (Yeasen, Shanghai, China) and colony formation. Three thousand cells were seeded into each well in a 96-well plate. The CCK-8 solution (10 µl) was added to 100 µl of culture media, and the optical density was measured at 450 nm. For colony formation assay, one thousand cells were seeded into each well in a 6-well plate for one week, then washed twice with PBS, fixed with 4% paraformaldehyde, stained with crystal violet, and the numbers of foci were counted for each well. Three independent experiments were performed.

### Transwell migration and invasion assays

For migration assay, 5 × 10^4^ cells were suspended in 200 µl of DMEM without serum and placed in the cell culture insert (8 µm pore size; BD Falcon, San Jose, CA) of a companion plate (BD Falcon) with a prewarmed culture medium containing 10% fetal bovine serum. For invasion assay, 1 × 10^5^ cells suspended in serum-free medium were seeded into the upper chamber coated with 1 µg/µl Matrigel (BD Biosciences, USA) of a 24-well transwell plate (8-μm pore size, Corning, NY, USA), then 600 μl DMEM with 10% FBS was added into the lower chamber. After incubation for an indicated period of time at 37 °C in 5% CO_2_, the migrating and invading cells on the outside of the upper chamber membrane were then fixed with 4% paraformaldehyde, stained with crystal violet, and counted under a light microscope (100× magnification) in eight randomly selected areas. Three independent experiments were performed.

### Statistical analysis

The expression of hub genes was assessed using the Wilcoxon Signed rank test and Mann–Whitney U test. Experimental data were expressed as mean ± standard deviations from three independent experiments and were analyzed using SPSS software (21.0; SPSS, Inc, Chicago, IL). Continuous variables between two groups or among three groups were compared using the unpaired Student’s t-test or one-way analysis of variance (Bonferroni post hoc test) as appropriate. Categorical variables were compared using the Chi-square test or Fisher’s exact test as appropriate. All statistical tests were two-sided and a *P*-value < 0.05 was considered statistically significant.

## Results

### The role of EZH2 in HCC

**Fig. 2:**
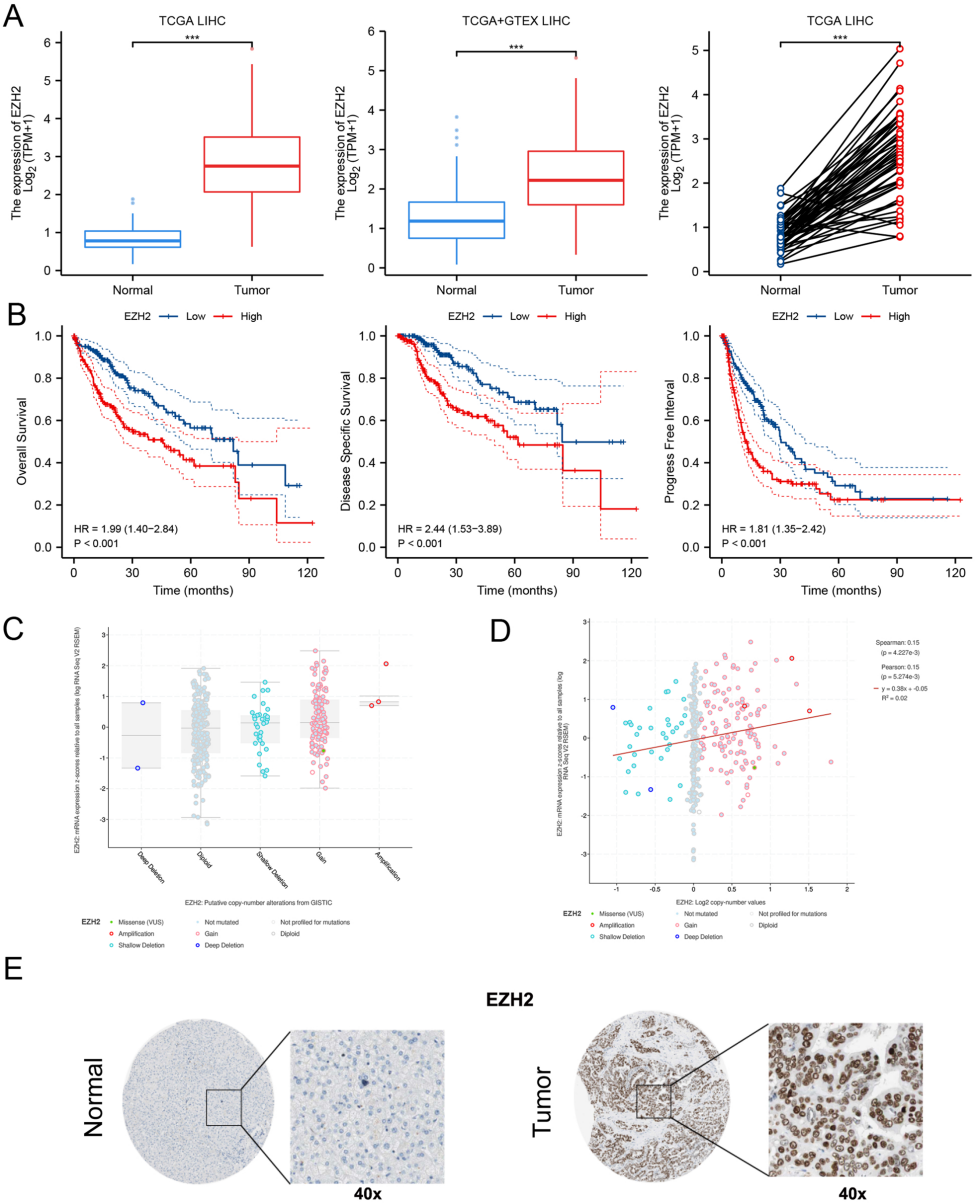
The Role of EZH2 in HCC. **A** The expression level of EZH2 in HCC tissues and paired/unpaired normal tissues through the TCGA database. **B** The overall survival, disease-specific survival, and progress-free interval of EZH2. **C** The putative copy-number alterations of EZH2 in HCC from cBioPortal database. **D** The relationship between the mRNA expression level and the copy number of EZH2 in HCC from cBioPortal database. **E** The immunohistochemistry analysis of EZH2 through the HPA database, and the result showed that EZH2 is mainly located in nuclear and highly expressed in tumor tissues compared with normal tissues. *P < 0.05, **P < 0.01, ***P < *0.001*

Previous studies have reported that EZH2 plays a crucial role in HCC. We hereby comprehensively discussed the important function of EZH2 in HCC through bioinformatics analysis [19–21]. The expression of EZH2 is frequently upregulated in HCC tissues compared to normal tissues (Fig. 2A). The related survival analysis of EZH2 showed that patients with high EZH2 expression have a worse overall survival (OS: HR = 1.99; 95% CI: 1.40–2.84; p < 0.001), disease-specific survival (DFS: HR = 2.44; 95% CI: 1.53–3.89; p < 0.001), and progression-free interval (PFI: HR = 1.81; 95% CI: 1.35–2.42; p < 0.001) (Fig. 2B). The association between EZH2 mRNA expression and copy number is shown in Fig. 2C and D), which suggested that the mRNA expression level of EZH2 is positively related to the copy number of EZH2 in HCC (R = 0.15, p = 4.227e−3). The protein expression of EZH2 in HCC and normal tissues were further evaluated through the HPA database. The immunohistochemistry of EZH2 showed that EZH2 is mainly located in the nuclear and highly expressed in tumor tissues compared with normal tissues (Fig. 2E). These results identified EZH2 as a prognostic factor, which promoted the progression of HCC. Future studies based on EZH2 may help explore more novel biomarkers and treatment strategies in HCC.

### Screening of DElncRNAs, DEmiRNAs, and DEmRNAs

**Fig. 3:**
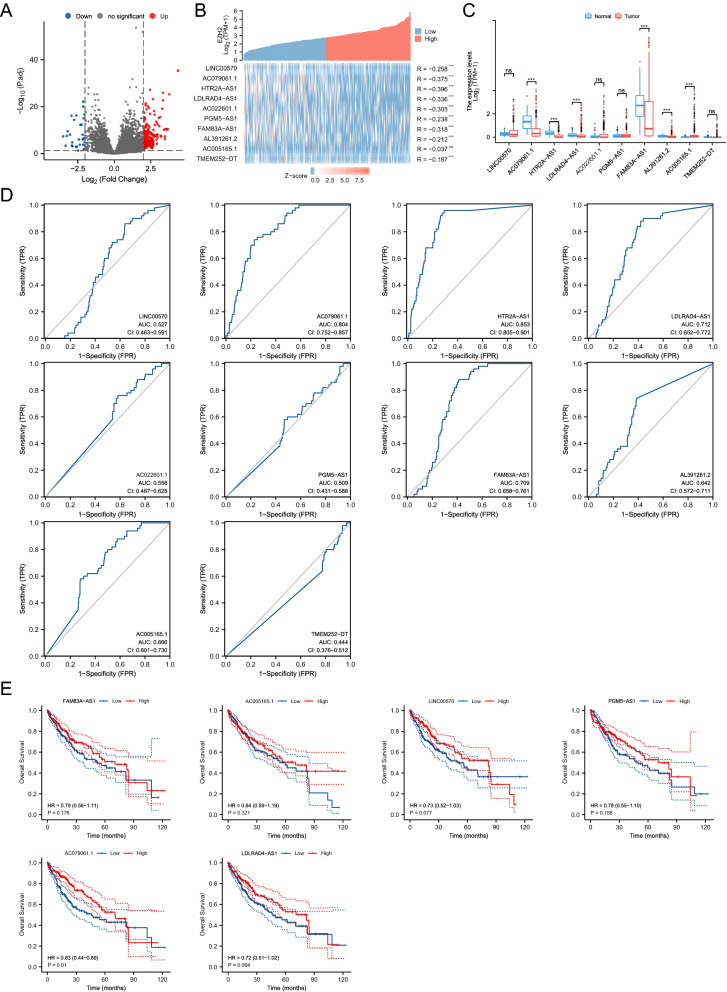
Screening of DElncRNAs. **A** The volcano map of 163 upregulated and 31 downregulated lncRNAs. **B** The co-expression analysis between the top 10 downregulated lncRNAs and EZH2. **C** The expression level of 10 lncRNAs between HCC tissues and normal tissues, and the result showed that 5 lncRNAs highly expressed in normal tissues, while 1 lncRNAs (AC005165.1) were highly expressed in tumor tissues. **D** The ROC analyses of top 10 downregulated lncRNAs. **E** The overall survival of 6 differentially expressed lncRNAs. *P < 0.05, **P < 0.01, ***P < *0.001*

Based on the expression of EZH2, the whole HCC cohort was divided into EZH2^high^ and EZH2^Low^ groups using median partitioning. The difference analysis was performed to obtain the differentially expressed lncRNAs (DElncRNAs). lncRNAs with |logFC|> 2 and p-value < 0.05 was considered as DElncRNAs. Then, 163 upregulated and 31 downregulated lncRNAs were visualized through the volcano map (Fig. 3A). The top 10 significant downregulated lncRNAs in order of p-value were selected for gene co-expression analysis, and the result showed that there is a strong negative correlation between 9 lncRNAs (LINC00570, AC079061.1, HTR2A-AS1, LDLRAD4-AS1, AC022601.1, PGM5-AS1, FAM83A-AS1, AL391261.2, and TMEM252-DT) and EZH2 (Fig. 3B). We assessed the expression level of 10 lncRNAs between HCC tissues and normal tissues, the result suggested that 5 lncRNAs (AC079061.1, HTR2A-AS1, LDLRAD4-AS1, FAM83A-AS1, and AL391261.2) were highly expressed in normal tissues, while 1 lncRNAs (AC005165.1) was highly expressed in tumor tissues (Fig. 3C). Then, ROC analyses were performed to assess the diagnostic value of these 10 lncRNAs. The results showed that only FAM83A-AS1, HTR2A-AS1, AC079061.1, and LDLRAD4-AS1 have a better diagnostic value in HCC, the area under curve (AUC) of these four lncRNAs were 0.709, 0.853, 0.804, and 0.712, respectively (Fig. 3D). Furthermore, survival analyses were used to evaluate the impact of lncRNAs on the outcome of HCC. Finally, the results suggested that AC079061.1 is the only lncRNA that can affect the prognosis of HCC. Patients with high AC079061.1 expression have a better outcome compared with patients with low AC079061.1 expression (HR = 0.63; 95% CI: 0.44–0.89; p = 0.01) (Fig. 3E).

**Fig. 4:**
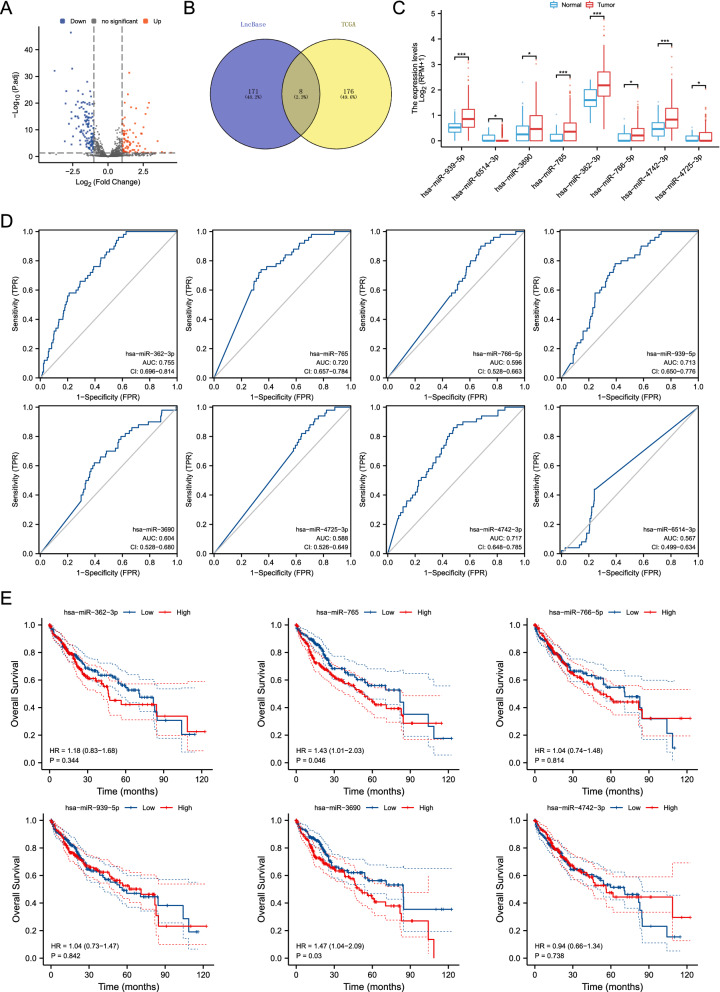
Screening of DEmiRNAs. **A** The volcano map of differentially expressed miRNAs in the TCGA database. **B** The overlapped miRNAs of between the LncBase predicted miRNAs and the DEmiRNAs in TCGA. **C** The expression levels of 8 overlapped miRNAs in tumor tissues and normal tissues, and the result showed that 2 miRNAs are downregulated in tumor tissues, and 6 miRNAs are elevated in tumor tissues. **D** The ROC analyses of 8 DEmiRNAs. **E** The overall survival of 6 elevated DEmiRNAs. *P < 0.05, **P < 0.01, ***P < *0.001*

Based on the ceRNA mechanism, potential miRNAs that may bind to DElncRNAs were first predicted using the LncBase v.2 database (http://carolina.imis.athena-innovation.gr/diana_tools/web/index.php?r=lncbasev2%2Findex-predicted). Meanwhile, the differentially expressed miRNAs between HCC tissues and normal tissues were obtained by the “limma” R packages. Finally, 8 miRNAs that may bind to DElncRNAs were identified in HCC by intersecting the LncBase predicted miRNAs with the DEmiRNAs in TCGA (Fig. 4A and B). The expression levels of these 8 miRNAs were confirmed in Fig. 4C, which showed that 2 miRNAs (hsa-miR-4725-3p and hsa-miR-6514-3p) were downregulated in tumor tissues, and 6 miRNAs (hsa-miR-939-5p, hsa-miR-3690, hsa-miR-765, hsa-miR-362-3p, hsa-miR-766-5p, and hsa-miR-4742-3p) were elevated in tumor tissues. Next, ROC analyses were performed to evaluate the diagnostic value of DEmiRNAs. The results suggested that hsa-miR-362-3p (AUC = 0.755), hsa-miR-765 (AUC = 0.720), hsa-miR-939-5p (AUC = 0.713), and hsa-miR-4742-3p (AUC = 0.717) have a better diagnostic value than hsa-miR-766-5p (AUC = 0.596), hsa-miR-3690 (AUC = 0.604), hsa-miR-4725-3p (AUC = 0.588), and hsa-miR-6514-3p (AUC = 0.567) (Fig. 4D). Furthermore, survival analyses were utilized to assess the prognostic value of DEmiRNAs. Results showed that only 2 miRNAs (hsa-miR-3690 (HR = 1.47, p = 0.03) and hsa-miR-765 (HR = 1.43, p = 0.046) affected the prognosis of HCC (Fig. 4E). Collectively, AC079061.1 may regulate the progression of HCC by binding to hsa-miR-765.

**Fig. 5:**
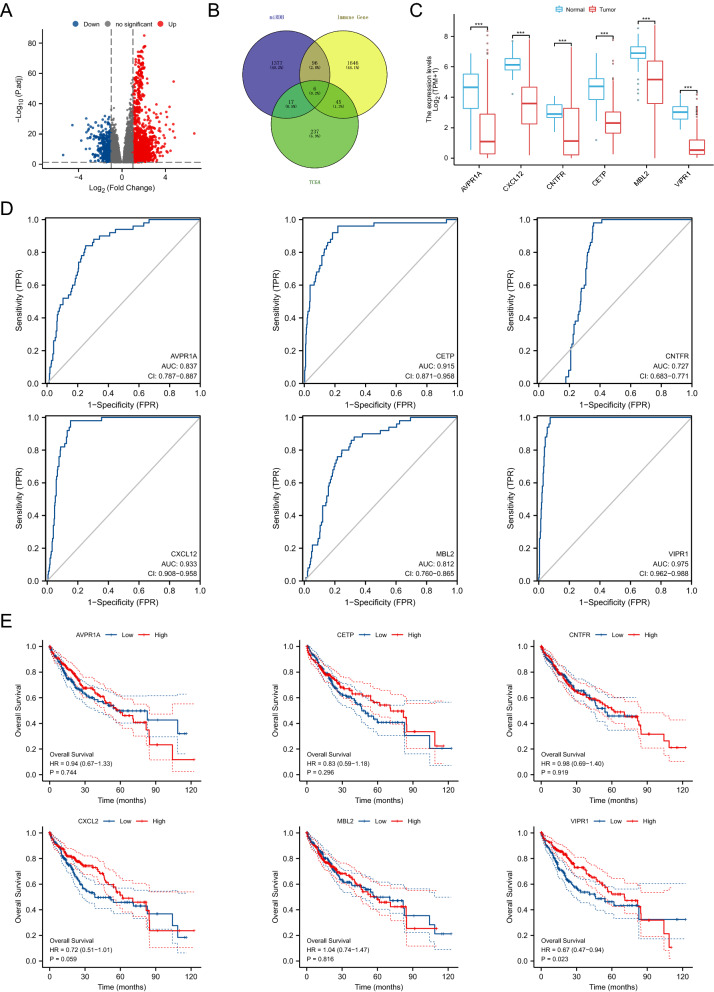
Screening of DEmRNAs. **A** The volcano map of differentially expressed mRNAs in the TCGA database. **B** The overlapped mRNAs of between the miRDB-predicted mRNAs, immune-related gene list and the DEmRNAs in TCGA. **C** The expression levels of 6 immune-related genes that may bind to the hsa-miR-765 in tumor tissues and normal tissues. **D** The ROC analyses of 6 DEmRNAs. **E** The overall survival of 6 DEmRNAs. *P < 0.05, **P < 0.01, ***P < *0.001*

In recent years, tumor immune microenvironment has been the focus of research and a hot topic. Many studies have suggested an inseparable relationship between tumorigenesis and the level of immune infiltration [22–25]. Hence, we report 6 immune-related genes that may bind to hsa-miR-765 by intersecting the miRDB-predicted miRNAs and immune-related gene list with DEmRNA (Fig. 5A and B). The expression level of differentially expressed immune-related genes (DEIRGs) was confirmed in Fig. 5C. The ROC and survival analysis results of 6 DEIRGs suggested that only VIPR1 (AUC = 0.975, HR = 0.67, p = 0.023) has both diagnostic and prognostic values in HCC (Fig. 5D and E).

### Correlation between lncRNA, miRNA, and mRNA

**Fig. 6:**
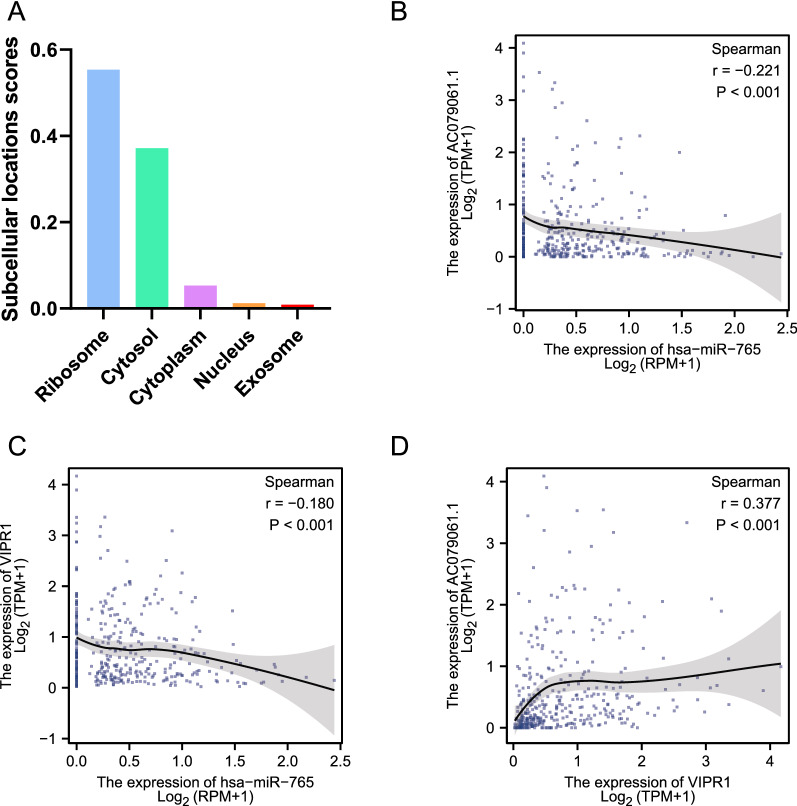
Correlation between lncRNA, miRNA, and mRNA. **A** Analyzed the subcellular localization of the lncRNA-AC079061.1 by the LNCipedia database. **B**, **C**, **D** The Spearman correlation analysis between the lncRNA- AC079061.1, hsa-miR-765, and VIPR1. *P < 0.05, **P < 0.01, ***P < *0.001*

To further explore the association between lncRNA- AC079061.1, hsa-miR-765, and VIPR1, we first analyzed the subcellular localization of the lncRNA- AC079061.1. The sequence of lncRNA-AC079061.1 was obtained from the LNCipedia database (https://lncipedia.org/db/transcript/lnc-EBAG9-9:1), and the sequence was imported into the lncLocator database to predict the subcellular location of lncRNA- AC079061.1. The results suggested that lncRNA- AC079061.1 was mainly located in the ribosome and cytosol (Fig. 6A). The Spearman correlation analysis was used to assess the correlation between lncRNA- AC079061.1, hsa-miR-765, and VIPR1 since the expression of these genes did not conform to normal distribution. Then, results showed a negative correlation between hsa-miR-765 and lncRNA- AC079061.1 or VIPR1. A positive correlation was also noted between lncRNA- AC079061.1 and VIPR1 (Fig. 6B–D).

### Immune infiltration analysis of lncRNA, miRNA, and mRNA

**Fig. 7:**
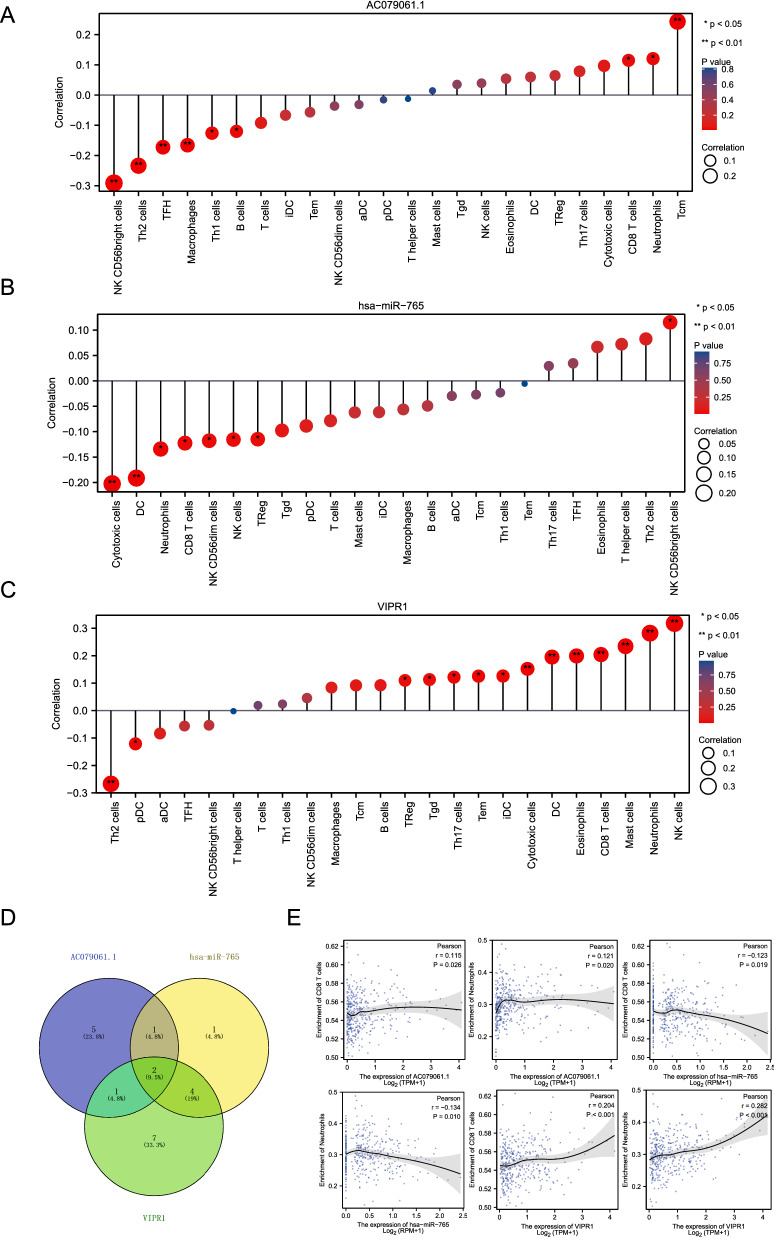
Immune infiltration analysis of lncRNA, miRNA, and mRNA. **A**, **B**, **C** Explored the correlation between immune infiltration and lncRNA- AC079061.1, hsa-miR-765, and VIPR1 through the ssGSEA algorithm in the “GSVA” R package. **D** Intersecting the correlated immune cells of lncRNA- AC079061.1, hsa-miR-765 and VIPR1. **E** Evaluated the correlation between CD8 T cells/Neutrophils and lncRNA- AC079061.1, hsa-miR-765, VIPR1 by the Pearson correlation analysis. *P < 0.05, **P < 0.01, ***P < *0.001*

Previous studies confirmed that immune cells were involved in the progression of HCC [26–28]. Thus, we explored the correlation between immune infiltration and these key genes. Initial results suggested that lncRNA-AC079061.1 was negatively correlated with NK CD56 bright cells, TFH, Th2 cells, macrophages, B cells, and Th1 cells, while positively correlated with neutrophils, Tcm, CD8 T cells (Fig. 7A). Subseqent results showed that hsa-miR-765 was negatively correlated with DC cells, cytotoxic cells, neutrophils, CD8 T cells, NK cells, NK CD56dim cells, and Treg, while positively correlated with NK CD56bright cells (Fig. 7B). VIPR1, an immune-related gene, was positively correlated with many immune cells such as NK cells, neutrophils, mast cells, CD8 T cells, and eosinophils, while negatively correlated with Th2 cells and pDC cells (Fig. 7C). Furthermore, we also analyzed the correlation between VIP or ADCYAP1 (VIPR1’s ligand) and the infiltration level of immune cells. Results showed that the VIP and ADCYAP1 were similar to VIPR1, which displayed a positive correlation with the infiltration level of NK cells, iDC, and TEM (Additional file 1: Fig. S1A–D), suggesting the possibility that altered expression of ligands and receptors may lead to the change in immune cell infiltration level and has a dramatic impact on HCC progression. Interestingly, we found that lncRNA AC079061.1, hsa-miR-765, and VIPR1 were all related to neutrophils and CD8 T cells. The correlation analysis showed that there is a positive relationship between lncRNA-AC079061.1/VIPR1 and CD8 T cells/Neutrophils (Fig. 7D and E). These results implied that the lncRNA-AC079061.1/hsa-miR-765/VIPR1 axis may influence the progression of HCC by regulating the immune infiltration level of neutrophils and CD8 T cells.

### Functional enrichment analysis of VIPR1

**Fig. 8:**
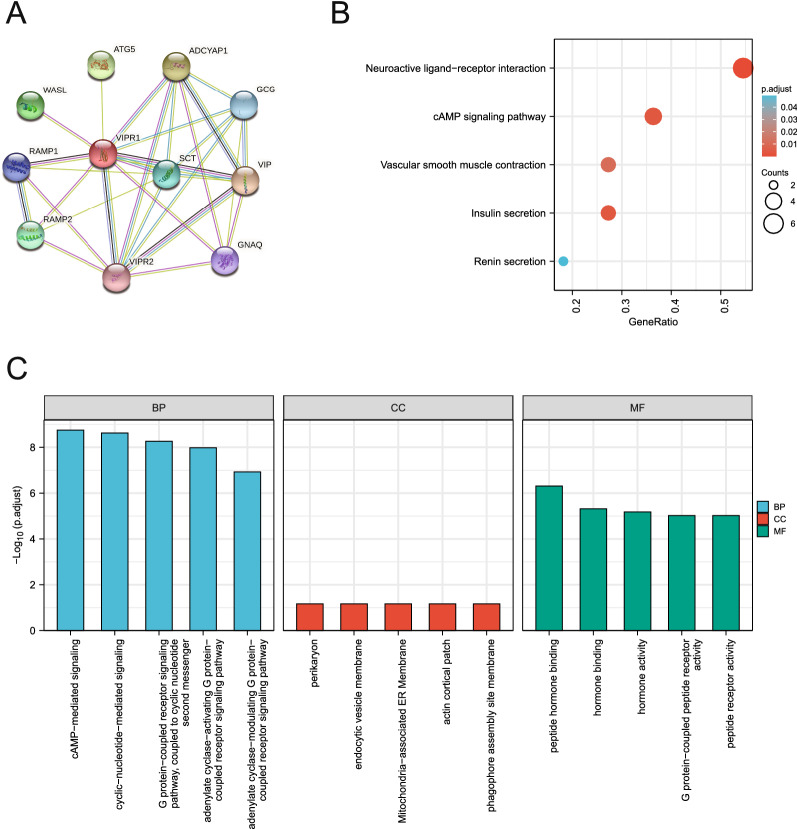
Functional enrichment analysis of VIPR1. **A** The interaction network between VIPR1 and related genes was obtained from the String database. **B**, **C** The KEGG and GO functional enrichment analyses of VIPR1 and its related genes

The potential mechanism of VIPR1 remains unclear and was therefore uploaded to the String database. Then, the interaction network between VIPR1 and related genes was obtained (Fig. 8A). The KEGG and GO functional enrichment analyses were performed to explore the potential VIPR1-related mechanism. The results showed that VIPR1 and its related genes were highly enriched in neuroactive ligand-receptor interaction (adjusted p-value = 1.38e−04), cAMP signaling pathway(adjusted p-value = 0.004), and vascular smooth muscle contraction (adjusted p-value = 0.011) (Fig. 8B). Subsequently, the most highly enriched GO BP terms were cyclic-nucleotide-mediated signaling (adjusted p-value = 2.38e−09), G protein-coupled receptor signaling pathway, coupled to cyclic nucleotide second messenger (adjusted p-value = 5.42e−09), and adenylate cyclase-activating G protein-coupled receptor signaling pathway (adjusted p-value = 1.05e−08). The GO CC terms showed that these genes may be located in the perikaryon (adjusted p-value = 0.068), endocytic vesicle membrane (adjusted p-value = 0.068), and Mitochondria-associated ER Membrane (adjusted p-value = 0.068). Furthermore, the highly enriched GO MF terms were peptide hormone binding (adjusted p-value = 4.89e−07), G protein-coupled peptide receptor activity (adjusted p-value = 9.55e−06), and peptide receptor activity (adjusted p-value = 9.55e−06) (Fig. 8C, Table 1).

**Table 1:** The top 5 KEGG and GO analysis results of VIPR1-related genes

Ontology	ID	Description	GeneRatio	BgRatio	p value	p.adjust	qvalue
BP	GO:0,019,933	cAMP-mediated signaling	7/11	187/18670	2.88e−12	1.77e−09	1.01e−09
BP	GO:0,019,935	cyclic-nucleotide-mediated signaling	7/11	215/18670	7.73e−12	2.38e−09	1.35e−09
BP	GO:0,007,187	G protein-coupled receptor signaling pathway, coupled to cyclic nucleotide second messenger	7/11	256/18670	2.64e−11	5.42e−09	3.09e−09
BP	GO: 0007189	adenylate cyclase-activating G protein-coupled receptor signaling pathway	6/11	139/18670	6.85e−11	1.05e−08	6.00e−09
BP	GO: 0007188	adenylate cyclase-modulating G protein-coupled receptor signaling pathway	6/11	221/18670	1.13e−09	1.18e−07	6.74e−08
CC	GO: 0043204	perikaryon	2/11	134/19717	0.002	0.068	0.044
CC	GO: 0030666	endocytic vesicle membrane	2/11	167/19717	0.004	0.068	0.044
CC	GO: 0044233	Mitochondria-associated ER Membrane	1/11	13/19717	0.007	0.068	0.044
CC	GO: 0030479	actin cortical patch	1/11	16/19717	0.009	0.068	0.044
CC	GO: 0034045	phagophore assembly site membrane	1/11	16/19717	0.009	0.068	0.044
MF	GO: 0017046	peptide hormone binding	4/11	49/17697	1.69e−08	4.89e−07	2.13e−07
MF	GO: 0042562	hormone binding	4/11	102/17697	3.33e−07	4.82e−06	2.10e−06
MF	GO: 0005179	hormone activity	4/11	122/17697	6.83e−07	6.61e−06	2.88e−06
MF	GO: 0008528	G protein-coupled peptide receptor activity	4/11	146/17697	1.40e−06	9.55e−06	4.16e−06
MF	GO: 0001653	peptide receptor activity	4/11	152/17697	1.65e−06	9.55e−06	4.16e−06
KEGG	hsa04080	Neuroactive ligand-receptor interaction	6/11	341/8076	2.09e−06	1.38e−04	1.19e−04
KEGG	hsa04024	cAMP signaling pathway	4/11	216/8076	1.42e−04	0.004	0.003
KEGG	hsa04911	Insulin secretion	3/11	86/8076	1.81e−04	0.004	0.003
KEGG	hsa04270	Vascular smooth muscle contraction	3/11	135/8076	6.83e−04	0.011	0.010
KEGG	hsa04924	Renin secretion	2/11	69/8076	0.004	0.050	0.043

To further explore the possible mechanisms of VIPR1, the top 100 VIPR1-related genes were downloaded from the GEPIA database (http://gepia.cancer-pku.cn/detail.php?gene=VIPR1) for another functional enrichment analysis (Additional file 1: Table S1.). More physiological processes such as complement activation, lectin pathway (adjusted p-value = 2.33e−04), positive regulation of vasculature development (adjusted p-value = 2.33e−04), cytokine receptor binding (adjusted p-value = 1.99e−04), receptor-ligand activity (adjusted p-value = 0.027) were obtained, suggesting that VIPR1 and its related genes play a key role in multiple mechanisms including the immune process. In summary, the results of these functional enrichment analyses suggested that VIPR1, as an immune-related gene, is involved in a variety of physiological processes (such as cytokine binding, inflammation-stimulation, and stress response) by regulating relevant genes, indicating the tremendous research value of VIPR1 in HCC.

### Methylation analysis of VIPR1 in HCC

Methylation is known to be aberrantly expressed in various forms of tumors. Meanwhile, it is capable of affecting not only the genetic phenotypes of cells but also the life span of humankind. There is a great potential for methylation in tumor diagnosis and treatments [29–31]. Therefore, multiple methods, as well as databases, were used to assess the correlation between VIPR1 and the progression of HCC. We first analyzed the correlation between VIPR1 expression and methyltransferases in HCC. As shown in Additional file 1: Fig. S2A, the results showed that there is a negative correlation between VIPR1 and DNMT1 (R = −0.150, p = 0.003) /DNMT3A (R = −0.250, p < 0.001). But no significant correlation was noted between VIPR1 and DNMT3B (R = −0.099, p = 0.057). Similarly, DiseaseMeth 2.0 database demonstrated that the methylation of VIPR1 was significantly lower in disease tissues compared with HCC normal tissues (p = 0.021**, **Additional file 1: Fig. S2B). Additionally, the CpG expression level in Additional file 1: Fig. S2C was obtained from the MethSurv platform, in which 8 methylation sites (cg27510539, cg12828331, cg23003500, cg10970409, cg04322483, cg22694480, cg27309542, cg23517013) in the DNA sequences of VIPR1 were found to be negatively associated with VIPR1 expression levels and 11 methylation sites (cg06783423, cg03791150, cg00328740, cg18559858, cg02061253, cg09475741, cg11484444, cg09996971, cg01919863, cg15066503, cg06290884) were found to be positively associated with VIPR1 expression level, analyzed through the MEXPRESS tool (Additional file 1: Fig. S2D). According to these results, altered methylation of VIPR1 may be an inducer of HCC.

### Prognostic analysis in HCC

**Fig. 9:**
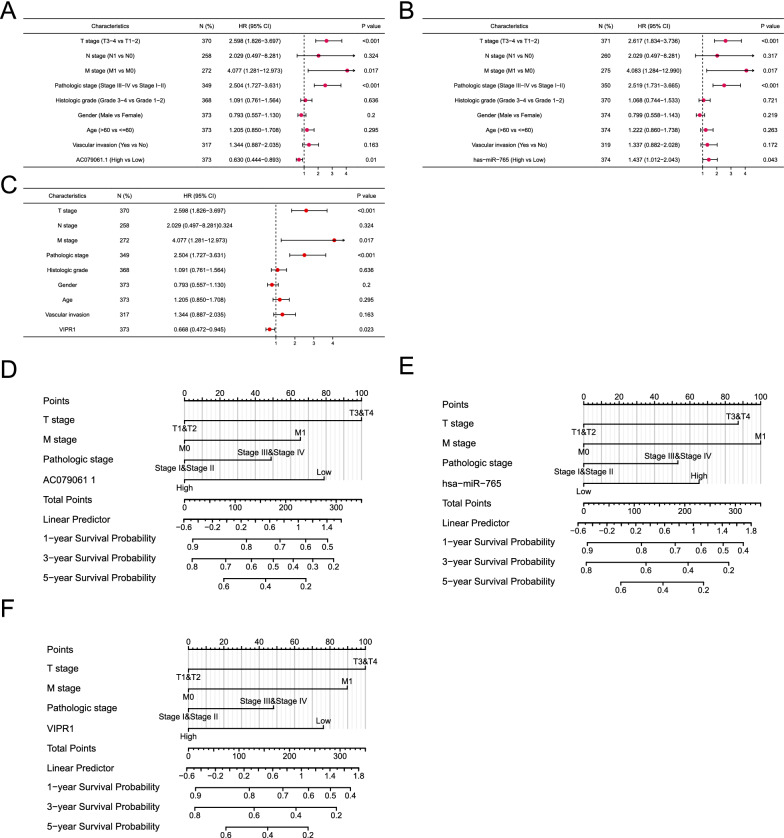
Prognostic analysis in HCC. **A**, **B**, **C** Univariate and multivariate cox regression analyses of lncRNA- AC079061.1, hsa-miR-765 and VIPR1. **D**, **E**, **F** Constructed the nomogram models to evaluate the relation between lncRNA AC079061.1/hsa-miR-765/VIPR1 and the overall survival of HCC

To assess the potential prognostic value of lncRNA AC079061.1, hsa-miR-765, and VIPR1 in HCC, univariate and multivariate cox regression analyses were performed. The results showed that lncRNA AC079061.1, hsa-miR-765, and VIPR1 as independent factors can affect the prognosis of HCC (Fig. 9A–C; Table 2, 3, 4). Then, we constructed prediction models to evaluate the relationship between lncRNA AC079061.1/hsa-miR-765/VIPR1 and the overall survival of HCC. The models were visualized by nomogram (Fig. 9D, E and F). All the results implied that the lncRNA AC079061.1/hsa-miR-765/VIPR1 axis acts as a prognostic indicator that may suppress the development of HCC.

**Table 2:** The univariate and multivariate analysis of lncRNA-AC079061.1 and clinicopathological features of HCC patients

Characteristics	Total(N)	Univariate analysis		Multivariate analysis	
		Hazard ratio (95% CI)	P value	Hazard ratio (95% CI)	P value
T stage	370				
T1 & T2	277	Reference			
T3 & T4	93	2.598 (1.826–3.697)	< 0.001	2.026 (0.274–14.988)	0.489
N stage	258				
N0	254	Reference			
N1	4	2.029 (0.497–8.281)	0.324		
M stage	272				
M0	268	Reference			
M1	4	4.077 (1.281–12.973)	0.017	1.581 (0.478–5.230)	0.453
Pathologic stage	349				
Stage I & Stage II	259	Reference			
Stage III & Stage IV	90	2.504 (1.727–3.631)	< 0.001	1.412 (0.192–10.368)	0.734
Histologic grade	368				
G1 & G2	233	Reference			
G3 & G4	135	1.091 (0.761–1.564)	0.636		
Gender	373				
Female	121	Reference			
Male	252	0.793 (0.557–1.130)	0.200		
Age	373				
≤ 60	177	Reference			
> 60	196	1.205 (0.850–1.708)	0.295		
Vascular invasion	317				
No	208	Reference			
Yes	109	1.344 (0.887–2.035)	0.163		
AC079061 1	373				
Low	186	Reference			
High	187	0.630 (0.444–0.893)	0.010	0.573 (0.365–0.899)	0.015

**Table 3:** The univariate and multivariate analysis of hsa-miR-765 and clinicopathological features of HCC patients

Characteristics	Total(N)	Univariate analysis		Multivariate analysis	
		Hazard ratio (95% CI)	P value	Hazard ratio (95% CI)	P value
T stage	371				
T1 & T2	279	Reference			
T3 & T4	93	2.617 (1.834–3.736)	< 0.001	1.844 (0.250–13.590)	0.548
N stage	260				
N0	256	Reference			
N1	4	2.051 (0.503–8.372)	0.317		
M stage	275				
M0	271	Reference			
M1	4	4.083 (1.284–12.990)	0.017	2.005 (0.614–6.544)	0.249
Pathologic stage	350				
Stage I & Stage II	261	Reference			
Stage III & Stage IV	90	2.519 (1.731–3.665)	< 0.001	1.452 (0.198–10.648)	0.713
Histologic grade	370				
G1&G2	232	Reference			
G3&G4	139	1.068 (0.744–1.533)	0.721		
Gender	374				
Female	119	Reference			
Male	256	0.799 (0.558–1.143)	0.219		
Age	374				
≤ 60	178	Reference			
> 60	196	1.222 (0.860–1.738)	0.263		
Vascular invasion	319				
No	208	Reference			
Yes	112	1.337 (0.882–2.028)	0.172		
hsa-miR-765	374				
Low	188	Reference			
High	187	1.437 (1.012–2.043)	0.043	1.579 (1.011–2.466)	0.044

**Table 4:** The univariate and multivariate analysis of VIPR1 and clinicopathological features of HCC patients

Characteristics	Total(N)	Univariate analysis		Multivariate analysis	
		Hazard ratio (95% CI)	P value	Hazard ratio (95% CI)	P value
T stage	370				
T1&T2	277	Reference			
T3&T4	93	2.598 (1.826–3.697)	< 0.001	2.025 (0.274–14.977)	0.489
N stage	258				
N0	254	Reference			
N1	4	2.029 (0.497–8.281)	0.324		
M stage	272				
M0	268	Reference			
M1	4	4.077 (1.281–12.973)	0.017	1.875 (0.574–6.128)	0.298
Pathologic stage	349				
Stage I&Stage II	259	Reference			
Stage III&Stage IV	90	2.504 (1.727–3.631)	< 0.001	1.404 (0.191–10.315)	0.739
Histologic grade	368				
G1&G2	233	Reference			
G3&G4	135	1.091 (0.761–1.564)	0.636		
Gender	373				
Female	121	Reference			
Male	252	0.793 (0.557–1.130)	0.200		
Age	373				
≤ 60	177	Reference			
> 60	196	1.205 (0.850–1.708)	0.295		
Vascular invasion	317				
No	208	Reference			
Yes	109	1.344 (0.887–2.035)	0.163		
VIPR1	373				
Low	186	Reference			
High	187	0.668 (0.472–0.945)	0.023	0.584 (0.377–0.904)	0.016

### Validation of lncRNA-AC079061.1/VIPR1 axis

**Fig. 10:**
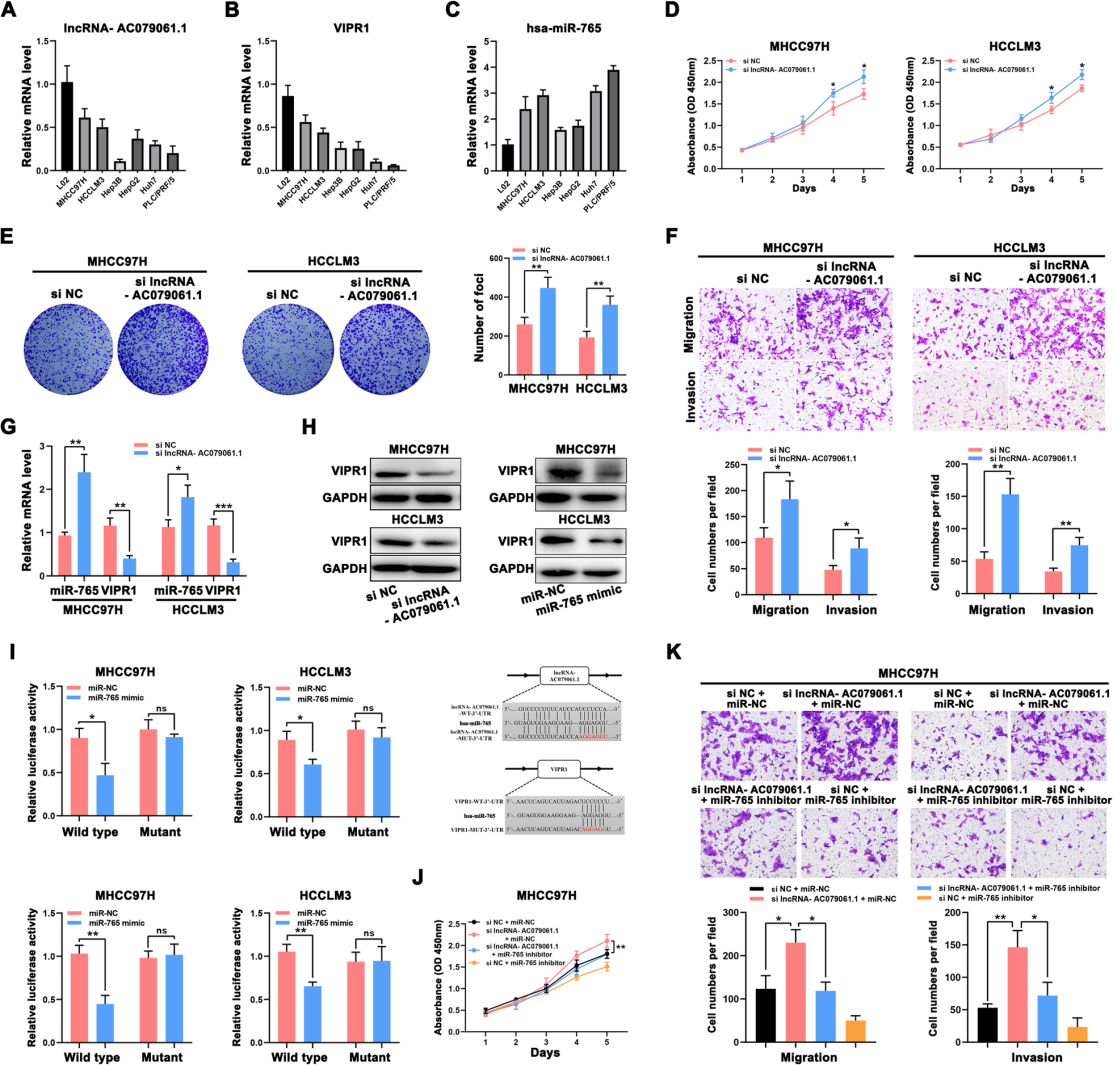
Validation of lncRNA-AC079061.1/VIPR1 axis. **A**, **B**, **C** The expression of lncRNA- AC079061.1, hsa-miR-765 and VIPR1 in the HCC cell lines and normal live cells. **D**, **E** Confirmed the proliferation of lncRNA- AC079061.1. by CCK-8 and colony formation assays. **F** Confirmed the invasion and migration ability of lncRNA- AC079061.1. by transwell assays. **G** Compared the mRNA level of hsa-miR-765 and VIPR1 between si-lncRNA- AC079061.1 group and control group in MHCC97H and HCCLM3 cells. **H** Detected the protein levels of VIPR1 transfected with lncRNA- AC079061.1 siRNA or miR-765 mimic. **I** The luciferase activity of wildtype-lncRNA-AC079061.1 or mutant-lncRNA- AC079061.1 (wildtype- VIPR1 or mutant-VIPR1) after co-transfection with miR-765 mimic in MHCC97H and HCCLM3 cells; Putative binding sites between miR-765 and lncRNA- AC079061.1 or VIPR1 were predicted via lncBase v2.0. **J**, **K** CCK-8, transwell migration, and invasion assays were performed in MHCC97H cells treated with si NC + miR-NC, si lncRNA-AC079061.1 + miR-NC, si lncRNA-AC079061.1 + miR-765 inhibitor, si NC + miR-765 inhibitor. *P < 0.05, **P < 0.01, ***P < *0.001*

To validate the feasibility of our comprehensive analysis, the expressions of these three genes were detected in the HCC cell lines and normal liver cells. We found that lncRNA and mRNA were frequently downregulated in HCC cells (Figs. 10A and B), while hsa-miR-765 was upregulated significantly in HCC cells (Fig. 10C). Then, we transfected the lncRNA- AC079061.1 siRNA into HCC cells, and the qRT-PCR was performed to confirm the stable downregulation of lncRNA- AC079061.1 (Additional file 1: Fig. S3). Moreover, to evaluate the relationship between lncRNA- AC079061.1 and the malignant phenotype of the tumor, a series of cellular function experiments was performed. The CCK-8 and colony formation assays suggested that the knockdown of lncRNA-AC079061.1 promoted the proliferation of HCC cells (Fig. 10D and E). Then, the transwell assays showed that the invasion and migration ability of si-lncRNA-AC079061.1 HCC cells were enhanced (Fig. 10F). These results indicated that lncRNA- AC079061.1 functions as an antioncogenic lncRNA to inhibit the proliferation, migration, and invasion of HCC cells in vitro. Additionally, to further verify the lncRNA-AC079061.1/VIPR1 axis, we first detected the expression level of hsa-miR-765 and VIPR1 in si- lncRNA- AC079061.1 HCC cells. Results showed that hsa-miR-765 is elevated in si- lncRNA- AC079061.1 cells compared with control cells (Fig. 10G), while the mRNA (Fig. 10G) and protein expression (Fig. 10H) of VIPR1 was decreased as the downregulation of lncRNA- AC079061.1 or upregulation of miR-765. Then, we performed dual-luciferase reporter assays in HCC cells and found that the luciferase activity was significantly downregulated after increasing the hsa-miR-765 expression in MHCC97H and HCCLM3 cells transfected with lncRNA-AC079061.1 or VIPR1 wildtype vectors. These results suggested that lncRNA-AC079061.1 may regulate the expression of downstream gene VIPR1 through sponging hsa-miR-765 (Fig. 10I). Furthermore, we investigated the effects of miR-765 on lncRNA-AC079061.1-mediated antioncogenic processes in vitro. Upregulation of miR-765 inhibited si-lncRNA-AC079061.1 induced proliferation (Fig. 10J), migration, and invasion (Fig. 10K) in MHCC97H cells. Collectively, these results indicated that the lncRNA- AC079061.1/hsa-miR-765/VIPR1 axis may regulate the progression of HCC.

### Prediction of chemotherapeutic drugs

Clinically, chemotherapy remains one of the mainstream treatments for HCC. However, in recent years, HCC patients tend to develop tolerance to common chemotherapy drugs. Therefore, the exploration of novel drugs is currently an essential trend in research. By utilizing the GDSC and CTRP databases, we obtained the correlation between VIPR1 expression and drug sensitivity in pan-cancer. These results provided good reference for future research direction. The results showed that the key genes investigated in the present study did not only correlate with common drugs such as Gefitinib and Afatinib but also with some unreported drugs. These correlations may even help identify new therapeutic drugs for HCC (Additional file 1: Fig. S4).

## Discussion

Cancer-related mortality remains the leading cause of death and a major health burden in Asia. Approximately, 49.3% of new cancer cases are located in Asia. Interestingly, more than half of these cases were reported in China, indicating that tumors have always been a major health concern. According to the global cancer statistics, the number of new cases and deaths of liver cancer ranks 7th among all cancers in the United States [32]. Furthermore, regardless of gender, liver cancer has always been ranked top 10 leading causes of death [33]. The understanding of underlying molecular mechanisms in HCC is of great importance, especially in the early diagnosis and treatment of the disease. In the present study, bioinformatics analyses combined with experimental validation identified novel biomarkers for the diagnosis and treatment of HCC.

In recent years, the continuous improvements in bioinformatics have been well applied to the discovery of new research directions, new research targets, and therapeutic drugs [34–36]. Xu et al. identified several immune-related lncRNA signatures in HCC through bioinformatics technology [37]. Liang et al. found that methylation-related genes (CTF1, FZD8, PDK4, and ZNF334) affected the progression of HCC [38]. The present study identified the lncRNA- AC079061.1/hsa-miR-765/VIPR1 axis, providing great research value in HCC by employing bioinformatics technology.

In the present study, an EZH2-related ceRNA network that is associated with the prognosis of HCC was constructed. Previous studies confirmed EZH2 as an oncogene that accelerates the development of HCC and negatively regulates the expression of immune checkpoint inhibitor PD-L1 in HCC [19, 20, 39]. Based on the function of EZH2, we screened for relevant differentially expressed lncRNAs, miRNAs, and mRNAs in HCC. Among all the DEGs, lncRNA- AC079061.1, hsa-miR-765, and VIPR1 potentially regulate the progression of HCC. hsa-miR-765 may bind to the 3’-UTR pf lncRNA- AC079061.1 and VIPR1, resulting in mutual regulation.

Interestingly, lncRNA- AC079061.1 has not been reported or studied before in HCC. Therefore, our study is the first to identify that this lncRNA has a function that suppresses HCC progression and the malignant phenotype of HCC. We also found that lncRNA- AC079061.1 is closely related to the prognosis of HCC.

In addition, it was reported that hsa-miR-765 is involved in various types of malignant tumors. Xie et al. verified that miR-765 promoted cell proliferation in HCC [40]. While Zhu et al. validated that LINC00994 repressed the malignant behaviors of pancreatic cancer cells through regulating the miR-765-3p/RUNX2 axis (which means that miR-765 accelerated the development of HCC) [41]. The oncogenic effect of miR-765 is not only limited to these two tumors, but also plays a similar role in esophageal squamous cell carcinoma [42], gastric cancer [43], and osteosarcoma [44].

For VIPR1, there are few relevant studies in HCC. However, low expression of VIPR1 has been shown to have an adverse prognostic impact on HCC [45]. However, it is not enough to study the role of VIPR1 in HCC alone, Zhao et al. also pointed out that VIPR1 has been confirmed to play the same role in lung adenocarcinoma [46]. These studies revealed the good theoretical feasibility of VIPR1 in HCC.Then, DNA methylation is known to regulate gene transcription and silence tumor suppressors [47]. A previous study reported that the H3K27 deacetylation and promoter methylation affect the expression of VIPR1 [45]. Altogether, these findings suggested the antitumor effect of VIPR1 in HCC.

Combining the results of previous studies and corresponding prediction databases, lncRNA- AC079061.1, hsa-miR-765, and VIPR1 were collectively analyzed to investigate the influence on the outcome of HCC either as an independent prognostic factors or as a lncRNA- AC079061.1/hsa-miR-765/VIPR1 axis.

## Conclusion

In summary, the lncRNA- AC079061.1/hsa-miR-765/VIPR1 axis may be a novel biomarker in HCC through comprehensive analyses and experimental validation. The prognostic analyses also suggest that it may suppress the development of HCC. Conclusively, the novel axis has a great potential to explore the pathogenesis of HCC and provide future directions for new therapeutic targets and drugs.

## Supplementary Information

**Additional file 1: Table S1.** The functional enrichment analysis of 100 VIPR1-related genes. **Figure S1.** Immune infiltration analysis of VIP and ADCYAP1 A. C. Explored the correlation between immune infiltration and VIP and ADCYAP1 through the ssGSEA algorithm in the “GSVA” R package. B. D. Evaluated the correlation between Treg/Tem/NK/iDC cells and VIP, Tem/NK/iDC/Mast cells and ADCYAP by the Pearson correlation analysis. *P<0.05, **P<0.01, ***P<0.001. **Figure S2**. The methylation analysis of VIPR1 in HCC. A. The correlation between VIPR1 expression and methyltransferases in HCC. B. The methylation level of VIPR1 in disease tissues and normal tissues by DiseaseMeth 2.0 database. C. The CpG expression level of VIPR1 in HCC. D. The methylation site of VIPR1 DNA sequence association with gene expression was visualized using MEXPRESS. The expression of VIPR1 is illustrated by the blue line in the center of the plot. Pearson’s correlation coefficients and p values for methylation sites and query gene expression are shown on the right side. **Figure S3.** Confirmation of the stable downregulation of lncRNAAC079061.1 transfected with lncRNA- AC079061.1 siRNA into MHCC97H and HCCLM3 HCC cells by qRT-PCR. *P<0.05, **P<0.01, ***P<0.001. **Figure S4.** The correlation between drugs and the VIPR1 mRNA expression of HCC in CTRP and GDSC database

## Data Availability

All data generated or analyzed during this study are included in this article.

## Abbreviations

HCC: Hepatocellular carcinoma
TCGA: The Cancer Genome Atlas
DElncRNAs, DEmiRNAs, DEmRNAs: Differentially expressed lncRNAs/miRNAs/mRNAs
GO: Gene Ontology
KEGG: Kyoto Encyclopedia of Genes and Genomes
