# Supplementary material for: lncRNA-AC079061.1/VIPR1 axis may suppress the development of hepatocellular carcinoma: a bioinformatics analysis and experimental validation
Source: J Transl Med. 2022 Aug 29;20:379. doi: 10.1186/s12967-022-03573-7 (PMC9422102; doi:10.1186/s12967-022-03573-7)
Supplement: Supplementary file 1 — Additional file 1: Table S1. The functional enrichment analysis of 100 VIPR1-related genes. Figure S1. Immune infiltration analysis of VIP and ADCYAP1 A. C. Explored the correlation between immune infiltration and VIP and ADCYAP1 through the ssGSEA algorithm in the “GSVA” R package. B. D. Evaluated the correlation between Treg/Tem/NK/iDC cells and VIP, Tem/NK/iDC/Mast cells and ADCYAP by the Pearson correlation analysis. *P<0.05, **P<0.01, ***P<0.001. Figure S2. The methylation analysis of VIPR1 in HCC. A. The correlation between VIPR1 expression and methyltransferases in HCC. B. The methylation level of VIPR1 in disease tissues and normal tissues by DiseaseMeth 2.0 database. C. The CpG expression level of VIPR1 in HCC. D. The methylation site of VIPR1 DNA sequence association with gene expression was visualized using MEXPRESS. The expression of VIPR1 is illustrated by the blue line in the center of the plot. Pearson’s correlation coefficients and p values for methylation sites and query gene expression are shown on the right side. Figure S3. Confirmation of the stable downregulation of lncRNAAC079061.1 transfected with lncRNA- AC079061.1 siRNA into MHCC97H and HCCLM3 HCC cells by qRT-PCR. *P<0.05, **P<0.01, ***P<0.001. Figure S4. The correlation between drugs and the VIPR1 mRNA expression of HCC in CTRP and GDSC database [file 12967_2022_3573_MOESM1_ESM.pdf]

Supplementary Table . The functional enrichment analysis of 100 VIPR1-related genes

ONTOLOGY	ID	Description	GeneRatio	BgRatio	pvalue	p.adjust	qvalue
**BP**	GO:0001867	complement activation, lectin pathway	4/73	13/18670	1.50e-07	2.33e-04	1.83e-04
**BP**	GO:1904018	positive regulation of vasculature development	9/73	230/18670	2.74e-07	2.33e-04	1.83e-04
**BP**	GO:0045766	positive regulation of angiogenesis	8/73	204/18670	1.30e-06	7.36e-04	5.79e-04
**BP**	GO:0007188	adenylate cyclase-modulating G protein-coupled receptor signaling pathway	8/73	221/18670	2.36e-06	0.001	7.90e-04
**BP**	GO:1901342	regulation of vasculature development	10/73	422/18670	5.46e-06	0.002	0.001
**CC**	GO:0005581	collagen trimer	6/79	87/19717	1.33e-06	2.13e-04	1.95e-04
**CC**	GO:0062023	collagen-containing extracellular matrix	10/79	406/19717	4.99e-06	3.99e-04	3.65e-04
**CC**	GO:0030014	CCR4-NOT complex	2/79	17/19717	0.002	0.100	0.091
**CC**	GO:0099056	integral component of presynaptic membrane	3/79	73/19717	0.003	0.100	0.091
**CC**	GO:0046930	pore complex	2/79	23/19717	0.004	0.100	0.091
**MF**	GO:0030246	carbohydrate binding	9/70	271/17697	1.17e-06	1.99e-04	1.55e-04
**MF**	GO:0005126	cytokine receptor binding	9/70	286/17697	1.82e-06	1.99e-04	1.55e-04
**MF**	GO:0005201	extracellular matrix structural constituent	7/70	163/17697	3.64e-06	2.66e-04	2.07e-04
**MF**	GO:0048029	monosaccharide binding	4/70	75/17697	2.21e-04	0.012	0.009
**MF**	GO:0048018	receptor ligand activity	8/70	482/17697	6.14e-04	0.027	0.021

Supplementary Figure legend

Supplementary Figure 1 Immune infiltration analysis of VIP and ADCYAP1

A. C. Explored the correlation between immune infiltration and VIP and ADCYAP1 through the ssGSEA algorithm in the "GSVA" R package. **B. D.** Evaluated the correlation between Treg/Tem/NK/iDC cells and VIP, Tem/NK/iDC/Mast cells and ADCYAP by the Pearson correlation analysis. *$*P<0.05$, $**P<0.01$, $***P<0.001$.*

Supplementary Figure 2 The methylation analysis of VIPR1 in HCC

A. The correlation between VIPR1 expression and methyltransferases in HCC. **B.** The methylation level of VIPR1 in disease tissues and normal tissues by DiseaseMeth 2.0 database. **C.** The CpG expression level of VIPR1 in HCC. **D.** The methylation site of VIPR1 DNA sequence association with gene expression was visualized using MEXPRESS. The expression of VIPR1 is illustrated by the blue line in the center of the plot. Pearson's correlation coefficients and p values for methylation sites and query gene expression are shown on the right side.

Supplementary Figure 3 Confirmation of the stable downregulation of lncRNA-AC079061.1 transfected with lncRNA- AC079061.1 siRNA into MHCC97H and HCCLM3 HCC cells by qRT-PCR. *$*P<0.05$, $**P<0.01$, $***P<0.001$.*

Supplementary Figure 4

The correlation between drugs and the VIPR1 mRNA expression of HCC in CTRP and GDSC database.

.

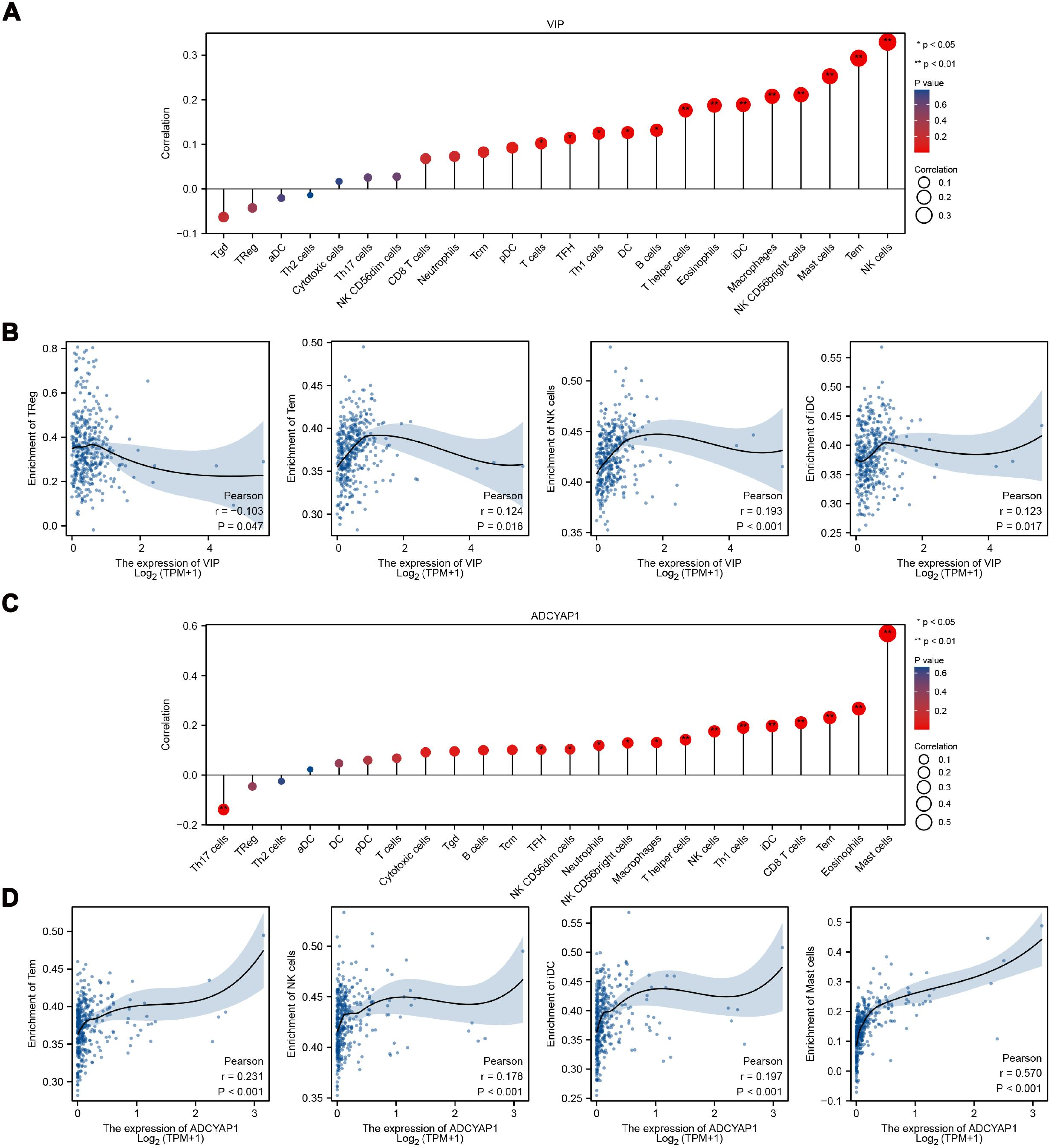
A
VIP
* p < 0.05
** p < 0.01
P value
0.6
0.4
0.2
Correlation
0.1
0.2
0.3
Correlation
Tgd
TReg
aDC
Th2 cells
Cytotoxic cells
Th17 cells
NK CD56dim cells
CD8 T cells
Neutrophils
Tcm
pDC
T cells
TFH
Th1 cells
DC
B cells
T helper cells
Eosinophils
iDC
Macrophages
NK CD56bright cells
Mast cells
Tem
NK cells
B
Enrichment of TReg
Pearson
r = −0.103
P = 0.047
Enrichment of Tem
Pearson
r = 0.124
P = 0.016
Enrichment of NK cells
Pearson
r = 0.193
P < 0.001
Enrichment of iDC
Pearson
r = 0.123
P = 0.017
The expression of VIP
Log2 (TPM+1)
C
ADCYAP1
* p < 0.05
** p < 0.01
P value
Correlation
Th17 cells
TReg
Th2 cells
aDC
DC
pDC
T cells
Cytotoxic cells
Tgd
B cells
Tcm
TFH
NK CD56dim cells
Neutrophils
NK CD56bright cells
Macrophages
T helper cells
NK cells
Th1 cells
iDC
CD8 T cells
Tem
Eosinophils
Mast cells
D
Enrichment of Tem
Pearson
r = 0.231
P < 0.001
Enrichment of NK cells
Pearson
r = 0.176
P < 0.001
Enrichment of iDC
Pearson
r = 0.197
P < 0.001
Enrichment of Mast cells
Pearson
r = 0.570
P < 0.001
The expression of ADCYAP1
Log2 (TPM+1)

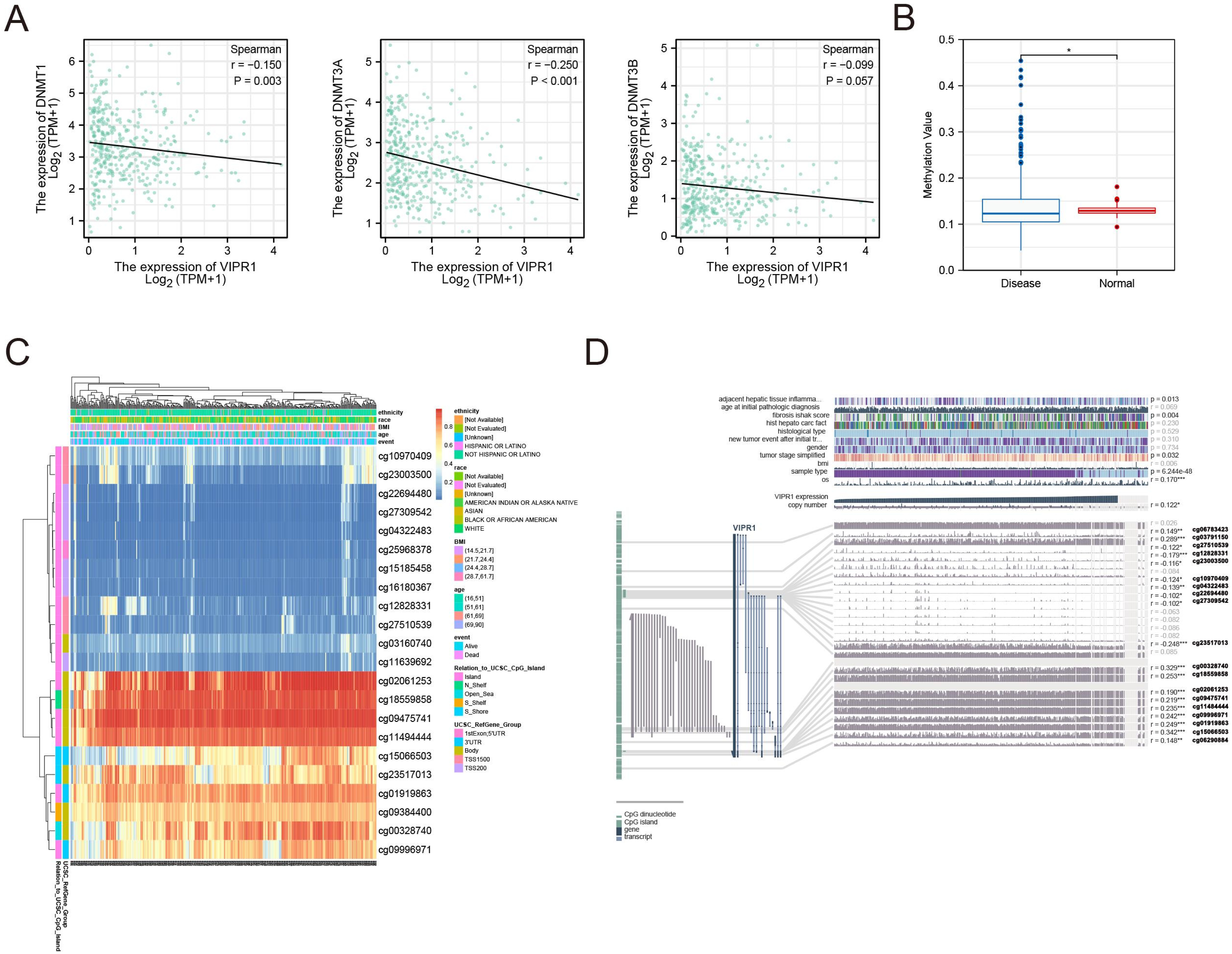
A
Spearman
r = −0.150
P = 0.003
The expression of DNMT1
Log2 (TPM+1)
The expression of VIPR1
Log2 (TPM+1)
Spearman
r = −0.250
P < 0.001
The expression of DNMT3A
Log2 (TPM+1)
The expression of VIPR1
Log2 (TPM+1)
Spearman
r = −0.099
P = 0.057
The expression of DNMT3B
Log2 (TPM+1)
The expression of VIPR1
Log2 (TPM+1)
B
Methylation Value
Disease
Normal
C
ethnicity
race
BMI
age
event
cg10970409
cg23003500
cg22694480
cg27309542
cg04322483
cg25968378
cg15185458
cg16180367
cg12828331
cg27510539
cg03160740
cg11639692
cg02061253
cg18559858
cg09475741
cg11494444
cg15066503
cg23517013
cg01919863
cg09384400
cg00328740
cg09996971
UCSC_RefGene_Group
Relation_to_UCSC_CpG_Island
ethnicity
[Not Available]
[Not Evaluated]
[Unknown]
HISPANIC OR LATINO
NOT HISPANIC OR LATINO
race
[Not Available]
[Not Evaluated]
[Unknown]
AMERICAN INDIAN OR ALASKA NATIVE
ASIAN
BLACK OR AFRICAN AMERICAN
WHITE
BMI
(14.5,21.7]
(21.7,24.4]
(24.4,28.7]
(28.7,61.7]
age
(16,51]
(51,61]
(61,69]
(69,90]
event
Alive
Dead
Relation_to_UCSC_CpG_Island
Island
N_Shelf
Open_Sea
S_Shelf
S_Shore
UCSC_RefGene_Group
1stExon;5'UTR
3'UTR
Body
TSS1500
TSS200
D
adjacent hepatic tissue inflamma...
age at initial pathologic diagnosis
fibrosis ishak score
hist hepato carc fact
histological type
new tumor event after initial tr...
gender
tumor stage simplified
bmi
sample type
os
p = 0.013
r = 0.069
p = 0.004
p = 0.230
p = 0.529
p = 0.310
p = 0.734
p = 0.032
r = 0.006
p = 6.244e-48
r = 0.170***
VIPR1 expression
copy number
r = 0.122*
VIPR1
r = 0.026
r = 0.149** cg06783423
r = 0.289*** cg03791150
r = -0.122* cg27510539
r = -0.179*** cg12828331
r = -0.116* cg23003500
r = -0.084
r = -0.124* cg10970409
r = -0.139** cg04322483
r = -0.102* cg22694480
r = -0.102* cg27309542
r = -0.063
r = -0.082
r = -0.086
r = -0.082
r = -0.248*** cg23517013
r = 0.085
r = 0.329*** cg00328740
r = 0.253*** cg18559858
r = 0.190*** cg02061253
r = 0.219*** cg09475741
r = 0.235*** cg11484444
r = 0.242*** cg09996971
r = 0.249*** cg01919863
r = 0.342*** cg15066503
r = 0.148** cg06290884
CpG dinucleotide
CpG island
gene
transcript

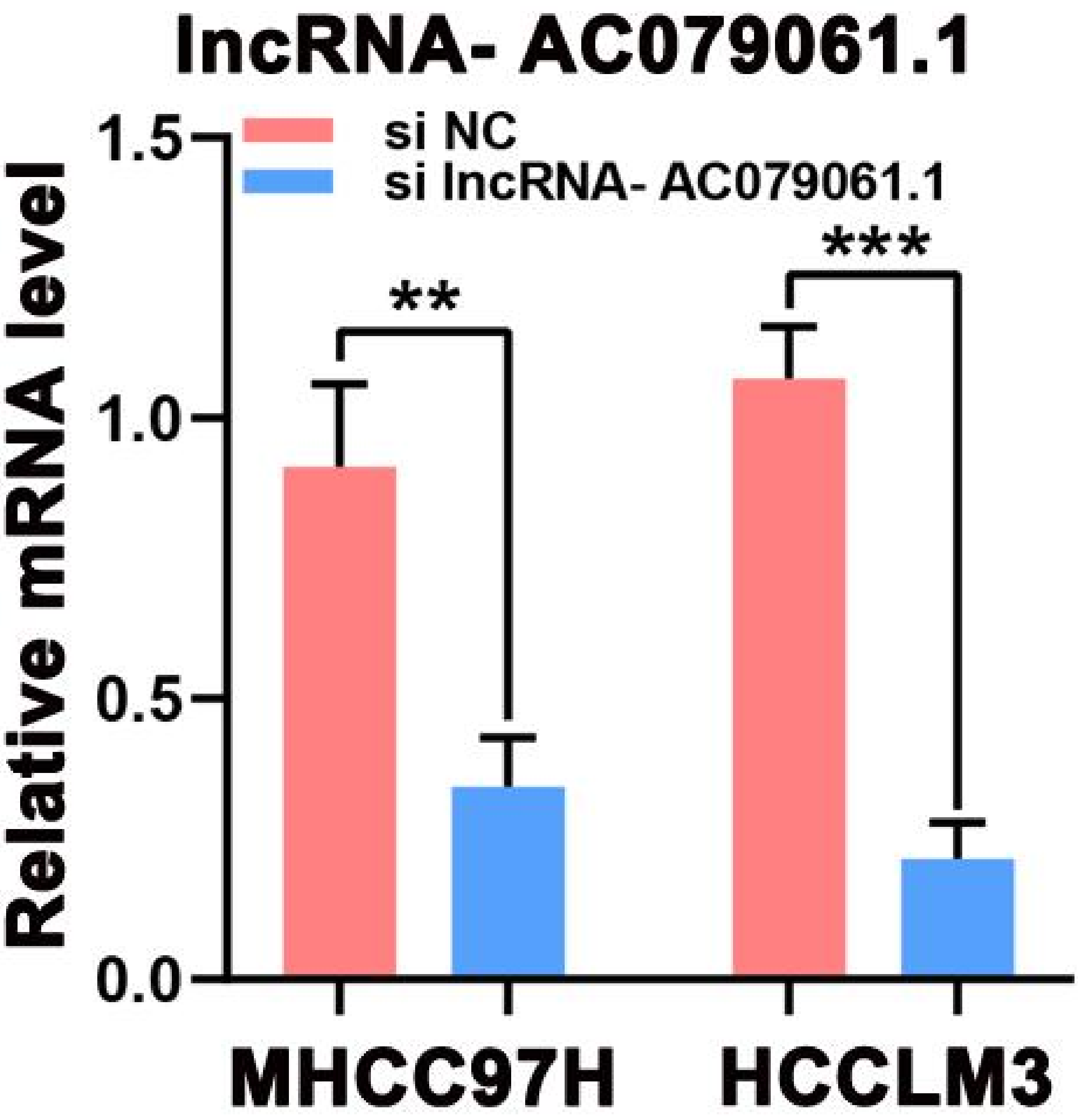
lncRNA- AC079061.1
si NC
si lncRNA- AC079061.1
Relative mRNA level
1.5
1.0
0.5
0.0
**
***
MHCC97H
HCCLM3

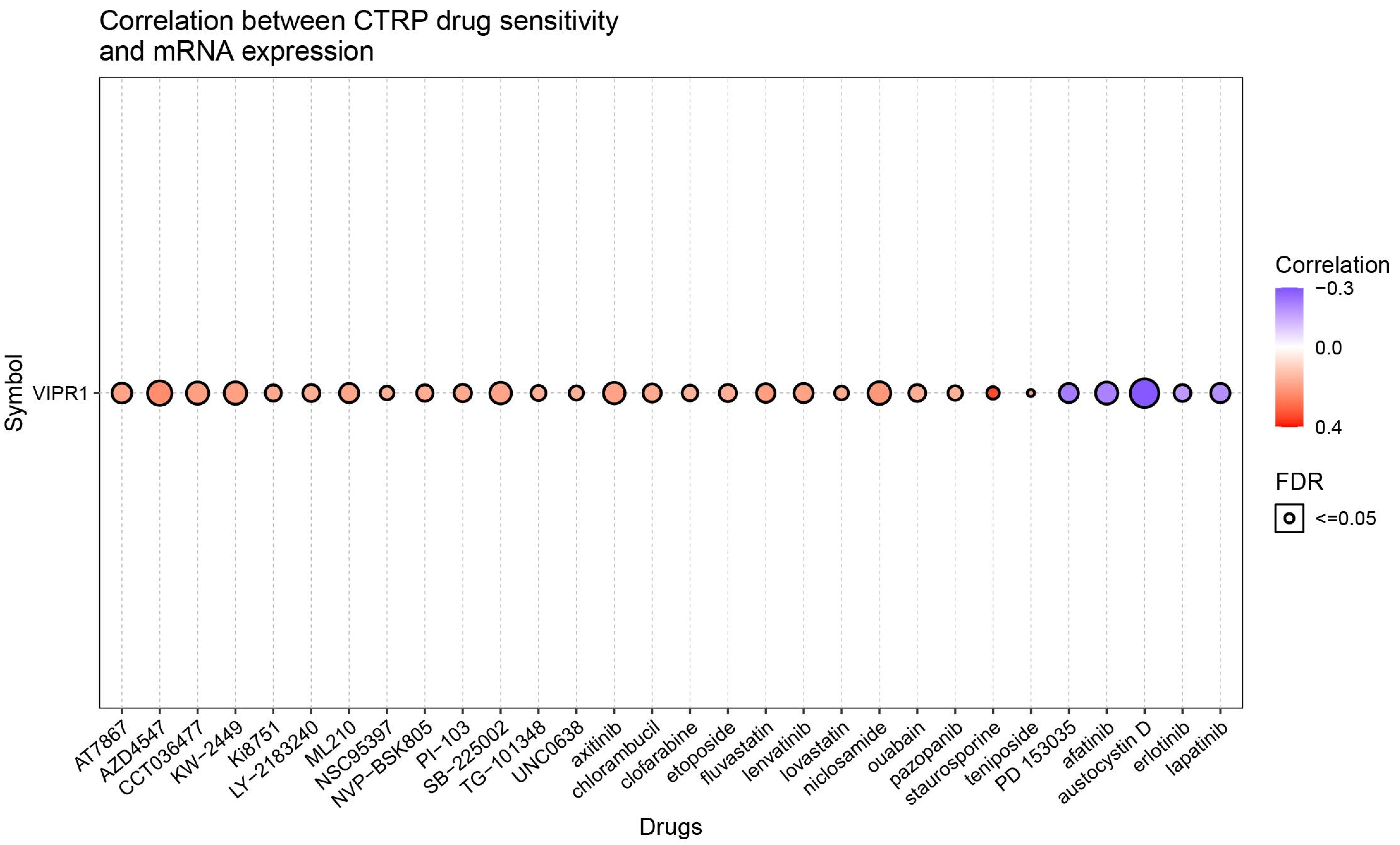
Correlation between CTRP drug sensitivity and mRNA expression
Symbol
VIPR1
Drugs
AT7867
AZD4547
CCT036477
KW−2449
Ki8751
LY−2183240
ML210
NSC95397
NVP−BSK805
PI−103
SB−225002
TG−101348
UNC0638
axitinib
chlorambucil
clofarabine
etoposide
fluvastatin
lenvatinib
lovastatin
niclosamide
ouabain
pazopanib
staurosporine
teniposide
PD 153035
afatinib
austocystin D
erlotinib
lapatinib
Correlation
−0.3
0.0
0.4
FDR
<=0.05

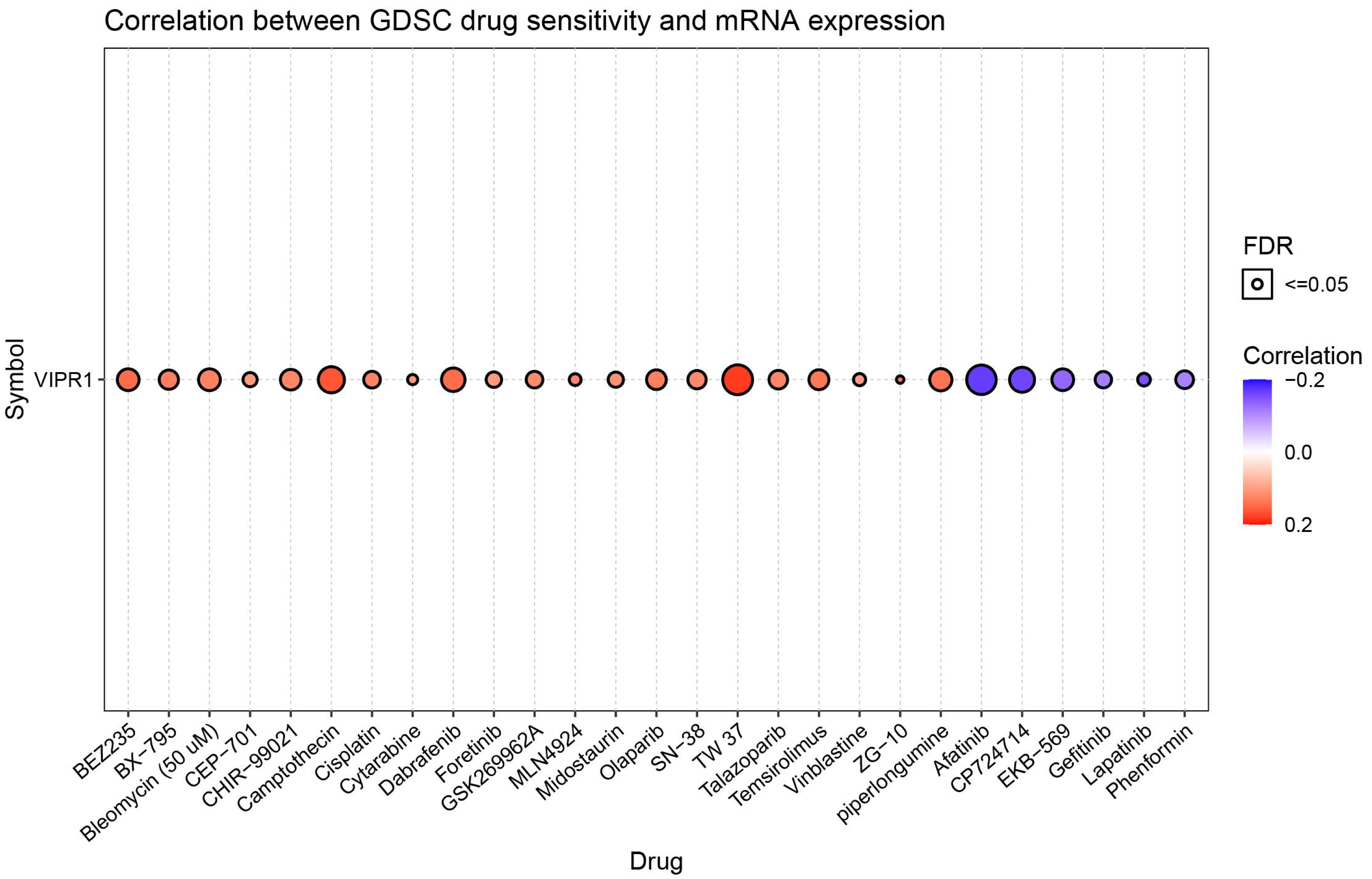
Correlation between GDSC drug sensitivity and mRNA expression
Symbol
VIPR1
Drug
BEZ235
BX−795
Bleomycin (50 uM)
CEP−701
CHIR−99021
Camptothecin
Cisplatin
Cytarabine
Dabrafenib
Foretinib
GSK269962A
MLN4924
Midostaurin
Olaparib
SN−38
TW 37
Talazoparib
Temsirolimus
Vinblastine
ZG−10
piperlongumine
Afatinib
CP724714
EKB−569
Gefitinib
Lapatinib
Phenformin
FDR
<=0.05
Correlation
−0.2
0.0
0.2
